# Commentary: seclusion and mechanical restraint of psychiatric patients in Israel - an update

**DOI:** 10.1186/s13584-019-0342-4

**Published:** 2019-10-14

**Authors:** Yoav Kohn, Igor Barash, Gadi Lubin

**Affiliations:** 1 Jerusalem Mental Health Center, Eitanim Psychiatric Hospital, 90972 DN Tzefon Yehuda, Israel; 2 Hebrew University-Hadassah School of Medicine, Jerusalem, Israel

**Keywords:** Mechanical restraint, Seclusion, Psychiatry

## Abstract

Recently, Miodownik et al. reported in this journal the results of a study on seclusion and mechanical restraint of psychiatric patients in Israel (Isr J Health Policy Res 8:9, 2019). The study was a retrospective examination over a year of one inpatient ward in a psychiatric hospital. They found negative associations between length of use of coercive measures and the diagnosis of schizophrenia, being single, and the presence of academic nurses. Positive associations were found between length of use of coercive measures and the use of antipsychotic medications, violence towards oneself, and the use of restraint compared to seclusion. Interesting and important as they are, these results were obtained from data gathered in 2014. As the authors note, since then there has been a dramatic change in the official policy of the Israeli Ministry of Health on this topic and in the practice of seclusion and mechanical restraint in Israel. This commentary reviews and comments on the current situation.

## Ministry of Health policy

Over the last decade, the issue of using coercive measures in psychiatry became a topic of a heated public debate. Both the media and patients advocate groups bitterly criticized the psychiatric system for using these measures too often and for the wrong reasons, claiming that by doing so they breach the rights of psychiatric patients while causing them unnecessary trauma [1]. Responding to that, on May 24, 2016 the Director General of the Ministry of Health appointed a steering committee to suggest a plan to reduce and eliminate mechanical restraint and seclusion of psychiatric patients in Israel. The committee submitted its report on May 29, 2017 [2]. It recommended restriction of these coercive measures to extreme situations of physical danger to oneself or to others, and to a minimal period of time. It recommended that mechanical restraint and seclusion of over 24 h be approved by a special external committee. It suggested forbidding the use of these measures if the only reason is the request of the patient. It also suggested not allowing mechanical restraint of minors under 13 years of age, and not allowing seclusion of such minors alone or for more than an hour. The committee set a goal of decreasing the use of coercive measures in Israel by 70% in the first year from implementation of its recommendations, an additional 60% in the second year and a further 60% in the third year. On the other hand, it acknowledged the need to train psychiatric staffs in alternative methods to treat aggressive behaviors. In accordance with the committee’s report, the Director General of the Ministry of Health issued a set of directives on April 1, 2018, implementing most of the above mentioned suggestions (apart from the ones dealing with minors) [3]. Elaborate instructions on the conditions in which coercive measures are allowed were specified, along with the mechanisms of ordering, executing and reporting them. In response, the Israel Medical Association instructed its members not to follow these directives, as no measures were taken to accompany them with increasing the number of medical and nursing staff and improving physical conditions in the wards. The professional organization of nurses in Israel took a similar position.

## Consequences of the new policy

Even though the debate over these new directives is still ongoing, and despite lack of support by the organizations representing physicians and nurses, the use of coercive measures in psychiatric hospitals is dramatically decreasing. In some hospitals this process started even before the change in policy of the Ministry of Health, as psychiatrists agreed that these measures should be kept only for situations of extreme danger to self and others, and should not be used as therapeutic tools, nor as a disciplinary action. For example, at Eitanim and Kfar Shaul, the psychiatric hospitals of the Jerusalem Mental Health Center (300 in-patient beds combined), the number of uses of mechanical restraints went down from 487 in 2015 to 64 in 2018. Similar trends have been reported in all other psychiatric hospitals in Israel. From discussions with colleagues, we know that at the same time in 8 out of the 10 psychiatric hospitals in Israel, there was a parallel increase in the number of violent acts against other patients and staff. In the other 2 psychiatric hospitals there was a slight decrease, which is attributed to less violent acts at the time of the restraint itself. It is reasonable to expect also that there was a parallel increase in the use of medications as an alternative to coercive measures, but we have no data to prove this. If there was an increase in use of medications, then a consequence could be increased prevalence of unwanted side effects. The increase in reports of violence that accompany the decreased use of coercive measures can be partly explained by the lack of a parallel increase in the numbers of physicians and nurses per ward. Other reasons that have been suggested by professionals in the field are the lack in training of staff as well as lack of improvement in physical conditions, all of which were supposed to accompany the new directives. Increasing the number of staff and special training in de-escalation techniques, for example, could help foster the use of alternative measures in dealing with aggressive and dangerous situations. Improving physical conditions is known to decrease the level of aggression in hospitalized psychiatric patients. A spacious and pleasant environment is unfortunately not common in psychiatric wards in Israel, which are many times located in old and under-maintained buildings. Such accompanying measures have been implemented in other countries such as the United Kingdom, where coercive measures are used less frequently and manual holding is preferred over mechanical restraint [4].

## The ethical dilemma

Using coercive measures in psychiatry violates the patient’s autonomy. A report to the Human Rights Council of the United Nations went to the extreme of defining it as “torture”, at least in some cases of prolonged restraint [5]. This view is unacceptable by most psychiatrists and authorities who see an ethical and moral demand to balance the autonomy of patients with the need to secure their safety, as well as the safety of others. The ethical value of beneficence dictates in these cases the use of non-consensual measures, especially when the free will and judgement of patients are hampered by disease. Indeed, in most western countries including the US, the Netherlands, Germany, Ireland, Norway, Finland and more, mechanical restraint and seclusion are being used, even though steps are taken to limit them [6–10].

The tension between the ethical values of autonomy and beneficence is inherent. The pendulum currently swings to the side of autonomy. This is influenced by pressure from the media and advocate groups. It should be noted that there is no parallel discussion, at least in Israel, about the similar use of mechanical restraint in non-psychiatric patients hospitalized in general hospitals and nursing homes. There is a wide use of mechanical restraints in older patients with dementia or delirium, who are being tied to their beds with non-standard equipment and no regulations concerning its use. It seems that some of the criticism about the use of coercive measures in mental health institutions stems from “anti-psychiatry” attitudes. Nevertheless, the government and professionals are aware of these changing attitudes, and psychiatrists share these views themselves to a certain extent and respond accordingly. For example, the former set of regulations on special orders for supervision, issued by the Division for Mental Health Services in Ministry of Health in 1992 [11] allowed the use of these measures in a broader set of conditions that are banned today. These included danger to property, severe psychomotor agitation (without danger to self and others), severe physical conditions, and an attempt to leave the ward or the hospital without permission or upon the request of the patient.

Paradoxically, the process of limiting the indications for the use of coercive measures, as manifested in the directives of the Ministry of Health, is occurring at the same time as there has been criticism of psychiatrists for not taking enough measures to ensure the safety of patients and others. In the last 5 years there was an increase of 450% in the total sum of compensation claims against psychiatric and geriatric state hospitals, from 2 million NIS in 2013 to 8.5 million NIS in 2018. In many of these cases the claims were for lack of protecting patients and others from violent behavior by other patients. Psychiatrists are being publicly criticized in cases where patients they treat or discharge (many times against medical advice) commit suicide or aggressive acts against others. In many instances, psychiatrists feel that they are being pushed towards “defensive medicine”. For example, the Ministry of Health leads a process of increase in reporting and reviewing extreme cases where patients are harmed or harm others by their acts. While the obvious reason for that is the need to better monitoring these cases, we know from colleagues that many psychiatrists feel attacked and, accordingly, respond with “defensive” measures.

## Conclusion

Psychiatrists and other mental health professionals face highly complex conditions in their daily work. They have to deal with life threating situations, conflicting ethical values, changing governmental policies, poor physical conditions of the work place and pressure from the public. In our opinion, one of the main findings of Miodownik et al. [12], which is relevant even in light of the changes that occurred since then, is the negative correlation between the length of the use of coercive measures and the presence on the wards of academic nurses (which usually means registered nurses with a baccalaureate degree). Education and training helps staff understand that physical restraint and seclusion should be used only to protect patients and others and not as therapeutic measures by themselves or as punishment. Indeed, in our hospitals we were able to achieve a decrease in the use of coercive measures without increasing the numbers of academic nurses. We and others have managed to do so through leadership of the medical management, internal discussions, and limited training of staff (funded mostly by internal resources). This process should be further strengthened by implementing the recommendation of the steering committee to the government to increase the number of staff, especially with highly educated professionals (e.g. academic nurses), to improve physical conditions, and to invest in training of staff. This would allow not only further reduction in mechanical restraint and seclusion but also the prevention of unwanted consequences, such as increase in violence of patients or the use of unneeded medication.

## Data Availability

Not applicable.
